# Evolution of Robustness in Growing Random Networks

**DOI:** 10.3390/e25091340

**Published:** 2023-09-15

**Authors:** Melvyn Tyloo

**Affiliations:** 1 Theoretical Division, Los Alamos National Laboratory, Los Alamos, NM 87545, USA; mtyloo@lanl.gov; 2 Center for Nonlinear Studies (CNLS), Los Alamos National Laboratory, Los Alamos, NM 87545, USA

**Keywords:** randomly growing networks, Kirchhoff index, robustness

## Abstract

Networks are widely used to model the interaction between individual dynamic systems. In many instances, the total number of units and interaction coupling are not fixed in time, and instead constantly evolve. In networks, this means that the number of nodes and edges both change over time. Various properties of coupled dynamic systems, such as their robustness against noise, essentially depend on the structure of the interaction network. Therefore, it is of considerable interest to predict how these properties are affected when the network grows as well as their relationship to the growth mechanism. Here, we focus on the time evolution of a network’s Kirchhoff index. We derive closed-form expressions for its variation in various scenarios, including the addition of both edges and nodes. For the latter case, we investigate the evolution where single nodes with one or two edges connecting to existing nodes are added recursively to a network. In both cases, we derive the relations between the properties of the nodes to which the new node connects along with the global evolution of network robustness. In particular, we show how different scalings of the Kirchhoff index can be obtained as a function of the number of nodes. We illustrate and confirm this theory via numerical simulations of randomly growing networks.

## 1. Introduction

Complex networks are broadly used to model interactions within natural and engineered systems [1,2,3]. They describe the interactions taking place between individual elements, such as the chemical bonds between atoms that form a molecules, or the communications transmitted between neighboring individuals in flocks of birds or vehicular platoons [4]. From their structure, important properties of coupled dynamic systems can be deduced, such as the intrinsic natural frequencies or the stability and robustness against external perturbations [5]. While in many instances both the structure of the coupling network and the number of interacting elements composing the system remain constant in time, this is typically not the case in a wide variety of coupled systems, such as social networks, vehicular platoon formation, swarming autonomous robots, animal collective behaviors, cells evolution, molecules interacting in chemical reactions, and more [6,7,8,9,10]. In all of these examples, when an element (commonly represented as a node) or an interaction (represented as an edge) is added to or removed from the system its overall dynamical properties are modified. In particular, both the steady states and the corresponding transient stability are affected by the evolution of the system. Therefore, it is an important task to predict how these properties change while the network evolves and to be able to anticipate potential instabilities. More specifically, if it is necessary to sequentially add agents to a system, it is important to understand how these interact with the existing units to ensure that stability is preserved, or at least not excessively hindered. This is the main question that we investigate in the present manuscript. Previous works have considered the evolution of network properties such as the degree distribution in random growing networks with preferential attachment [11,12] and the evolution of the Wiener index in random recursive trees [13]. In this manuscript, we investigate the time evolution of the *Kirchhoff index* [14,15,16], which has proven useful in chemistry [14,17,18] and networked dynamical systems [19,20]. For coupling networks that are not growing and which are static in time, the robustness of diffusively coupled oscillators has been directly related to the Kirchhoff index of the effective coupling network [19,21,22]; specifically, the larger the Kirchhoff index, the more important the fluctuations within the dynamic system. Consider a set of *N* oscillators, each with a continuous degree of freedom $x_{i}\in R$ that are diffusively coupled together and subjected to noise as follows:

(1) $$\begin{array}{c} {\dot{x}}_{i}=-\sum_{j=1}^{N}a_{ij}\left(x_{i}-x_{j}\right)+\eta_{i},\;i=1,\ldots ,N, \end{array}$$

where $a_{ij}=a_{ji}>0$ are the elements of the adjacency matrix encoding the undirected coupling network and $\eta_{i}$ represents uncorrelated white noise inputs, i.e., $\langle \eta_{i}\left(t\right)\eta_{j}\left(t^{\prime }\right)\rangle =\eta_{0}^{2}\;\delta_{ij}\;\delta \left(t-t\right)$. Then, the average variance in the long time limit is provided by [23]

(2) $$\begin{array}{c} \frac{1}{N}\sum_{j=1}^{N}\langle x_{j}^{2}\rangle =\frac{\eta_{0}^{2}}{2}Kf_{1}/N, \end{array}$$

with $Kf_{1}$ being the Kirchhoff index of the coupling network (see Section 2 for the definition). Similar relations can be obtained for deterministic perturbations that have a short correlation time [19]. Considering this direct connection between the global network index and the fluctuations of the dynamic system supported by the network, it is interesting to investigate how the Kirchhoff index evolves as the network grows. For the evolution of the network, we consider a simple growth algorithm in which a single new node that connects to existing nodes is added in each iteration. We derive the analytical expression for the time evolution of the Kirchhoff index for this scenario; in particular, when connecting the new node to the existing ones, we identify which of their nodal properties influence the scaling of the Kirchhoff index as a function of the total number of nodes. These properties can be used when adding new nodes in order to achieve different scalings for the Kirchhoff index as well as for the fluctuations.

The rest of this manuscript is organized as follows: in Section 2, we provide the definition of the Kirchhoff index and discuss the previously derived bounds; in Section 3 we consider growing networks and provide expressions for the time evolution of the Kirchhoff index when edges and nodes are added; finally, in Section 4 we provide our conclusions and future outlook.

## 2. Kirchhoff Index

### 2.1. Definitions

Consider a graph *G* (called a network in the following) made up of vertices *N* (called nodes in the following) and edges *M*. Each edge $\epsilon_{\left(ij\right)}$ between two nodes *i* and *j* has an associated weight $a_{ij}>0$. The network Laplacian matrix is commonly defined as *L*, with $L_{ij}=-a_{ij}$ if i≠j and there exists an edge between nodes *i* and *j*; otherwise, $L_{ij}=0$ and $L_{ii}=\sum_{k=1}^{N}a_{ik}$ for i=1,…,N. The Kirchhoff index ($Kf_{1}$) of an undirected network is provided by the sum of the effective resistance distances ($\Omega_{ij}$) between all the nodes [14]:

(3) $$Kf_{1}=\sum_{i<j}\Omega_{ij},$$

while the resistance distance between nodes *i* and *j* is defined by

(4) $$\Omega_{ij}={\left[L^{†}\right]}_{ii}-2{\left[L^{†}\right]}_{ij}+{\left[L^{†}\right]}_{jj},$$

where $L^{†}$ denotes the pseudo-inverse of the Laplacian matrix L of the network. Using the eigenvectors $u_{\alpha }$ and eigenvalues $\lambda_{1}=0<\lambda_{2}<\cdots <\lambda_{N}$ of L, we can conveniently rewrite the resistance distance $\Omega_{ij}=\sum_{\alpha >1}{\left(u_{\alpha ,i}-u_{\alpha ,j}\right)}^{2}/\lambda_{\alpha }$, which for the Kirchhoff index yields [24]

(5) $$Kf_{1}=N\sum_{\alpha >1}\frac{1}{\lambda_{\alpha }}=N\;\mathrm{Tr}\left[L^{†}\right].$$

Depending on the time scale of the noise input, the amplitude of the small fluctuations of diffusively coupled oscillators can be expressed in terms of the Kirchhoff index or its generalization, which reads [19]

(6) $$Kf_{p}=N\sum_{\alpha >1}\frac{1}{\lambda_{\alpha }^{p}}=N\;\mathrm{Tr}\left[{L^{†}}^{p}\right].$$

For network models for which the spectrum is known, the Kirchhoff index can be obtained analytically. For example, for a complete star network we have $Kf_{1}≅N,N^{2},N^{3}$ as the number of nodes *N* becomes large, while for a cycle network we have $Kf_{2}≅1,N^{2},N^{5}$. These network models prove useful below when we consider the limiting case of randomly growing networks. In the specific case where the network is a tree, the resistance distance is equal to the shortest path distance in the same network when all the weights on the edges have been replaced by their inverse weights. In such a situation, the Kirchhoff index is equal to the Wiener index [17], that is, it can be defined as the sum of all the shortest path distances in the network. In the following, we only discuss the Kirchhoff index, as we do not consider that growing networks need to be trees. From the resistance distance, it is possible to define a centrality measure that reads

(7) $$\begin{array}{c} C\left(i\right)={\left[\sum_{j=1}^{N}\Omega_{ij}/N\right]}^{-1}={\left[L_{ii}^{†}+Kf_{1}/N^{2}\right]}^{-1}, \end{array}$$

where C(i) is called the resistance centrality of node *i*.

### 2.2. Lower Bound on $Kf_{1}$

The Kirchhoff index has been extensively studied and many bounds have been derived depending on the number of nodes *N*, edges $n_{e}$, and other properties. Relevant for the following is the lower bound obtained by Zhou and Trinajstić [25], which states that for a connected network with N≥3, $n_{e}$ edges, and a maximum degree Δ,

(8) $$\begin{array}{c} Kf_{1}\left(N\right)\geq \frac{N}{1+\Delta }+\frac{N{\left(N-2\right)}^{2}}{2N_{e}-1-\Delta }. \end{array}$$

From this inequality, it can be concluded that as long as $n_{e}\propto N$, it is the case that $Kf_{1}/N$ scales by at least *N* when the number of nodes becomes large. This is the case in the growth algorithm we investigate below, in which a single new node is added at each iteration such that $n_{e}\propto N$. Therefore, the lowest scaling achievable for $Kf_{1}/N$ within our growing algorithm is linear in *N*.

## 3. Robustness of Growing Networks

Networks can grow in two ways: (i) new nodes are connected to the existing network nodes; and (ii) edges are added within the existing nodes. For (i), it is intuitive based on the examples in Section 2 and Equation (8) that the Kirchhoff index increases at least linearly with *N*. On the other hand, for (ii) it can be shown that the Kirchhoff index can only decrease by adding a new edge in the network. Adding one edge with corresponding weight $a_{kl}>0$ between nodes *k* and *l* is a rank-1 modification of the Laplacian matrix, i.e., $L\left(t+1\right)=L\left(t\right)+a_{kl}e_{kl}e_{kl}^{⊤}$, where ${\left[e_{kl}\right]}_{i}=\left(\delta_{ik}-\delta_{il}\right)\in R^{N_{t}}$, with $N_{t}$ being the number of nodes at iteration *t*. Therefore, if the Kirchhoff index at iteration *t* is Kf(t) then we can use the Sherman–Morrison–Woodbury formula [26,27] to obtain the Kirchhoff index at step t+1:

(9) $$\begin{array}{c} Kf_{k}\left(t+1\right)=Kf_{1}\left(t\right)-\frac{a_{kl}\mathrm{Tr}\left[L^{†}e_{kl}e_{kl}^{⊤}L^{†}\right]}{1+a_{kl}\Omega_{lk}\left(t\right)}\;=Kf_{1}\left(t\right)-\frac{a_{kl}\Omega_{kl}^{\left(2\right)}\left(t\right)}{1+a_{kl}\Omega_{kl}\left(t\right)}, \end{array}$$

where $\Omega_{kl}^{\left(2\right)}\left(t\right)=\sum_{\alpha >1}{\left(u_{\alpha ,i}-u_{\alpha ,j}\right)}^{2}/\lambda_{\alpha }^{2}$ is a semi-metric [21]. As both $\Omega_{kl}^{\left(2\right)}\left(t\right)$ and $\Omega_{kl}\left(t\right)$ are always positive, $Kf_{1}$ can only decrease when an edge is added to the existing network. Below, we discuss how the Kirchhoff index is modified when a single node together with *m* new edges is added to the existing network.

### 3.1. One New Node with a Single Connection (m=1)

**Figure 1:**
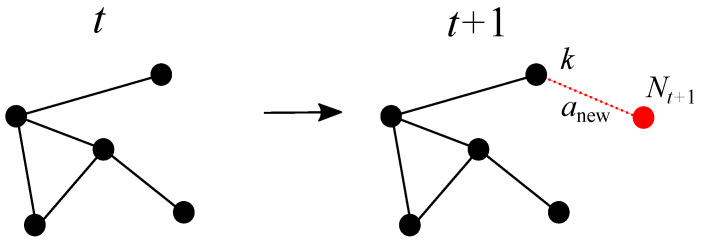
Evolution of the network from iteration *t* to t+1, where a new node (in red) connecting to a single existing node *k* (in black) has been added. The label of the new node is $N_{t+1}=N_{t}+1$. No new path is created within the existing nodes.

Below, we investigate the evolution of the Kirchhoff index for the growth process depicted in Figure 1. When a new node connects to a single existing one, if we start with a network that is a tree then the network will remain a tree as it grows. In addition, if the selected existing node is uniformly chosen at random, the resulting tree called a *random recursive tree*. In such a situation, the resistance distance can be replaced by the geodesic or shortest path distance (i.e., the Kirchhoff index by the Wiener index), as discussed in [13,28]. In general, however, we do not assume that the starting network is a tree. At iteration *t*, we has the Kirchhoff index $Kf_{1}\left(t\right)=\frac{1}{2}\sum_{i,j=1}^{N_{t}}\Omega_{ij}\left(t\right)$. If the new node at iteration t+1 is connected to node *k*, we have

(10) $$\begin{array}{ccc} Kf_{1}\left(t+1\right) & = & Kf_{1}\left(t\right)+\sum_{l=1}^{N_{t}}\Omega_{kl}\left(t\right)+\frac{N_{t}}{a_{\mathrm{new}}}, \end{array}$$

where $a_{k(N_{t+1})}=a_{\mathrm{new}}$ is the weight of the newly added edge between nodes *k* and $N_{t+1}$. In this simple case, it can be observed that the modification of the Kirchhoff index is provided by the sum of the resistance distances from node *k* to all the other already existing nodes in the network plus $N_{t}$ times the resistance of the newly added edge; see Figure 1. The less central node *k* is in terms of its resistance distances from the existing nodes, the more the Kirchhoff index grows. As expected, $Kf_{1}\left(t\right)$ only increases with the number of iterations, as no new path is created within the existing nodes. If the node to which the new node connects is uniformly selected at random among the existing ones with each new iteration, then on average the Kirchhoff index will increase as follows:

(11) $$\begin{array}{ccc} \langle Kf_{1}\left(t+1\right)\rangle & = & \langle Kf_{1}\left(t\right)\rangle \left(1+\frac{2}{N_{t}}\right)+\frac{N_{t}}{a_{\mathrm{new}}} \end{array}\;$$

$$\begin{array}{ccc} & = & \frac{\left(N_{0}+t+1\right)}{a_{\mathrm{new}}}[\frac{a_{\mathrm{new}}\frac{Kf_{1}\left(0\right)\left(N_{0}+2\right)}{N_{0}}\left(N_{0}+t+2\right)-2\left(N_{0}+2\right)\left(t+1\right)}{\left(N_{0}+1\right)\left(N_{0}+2\right)} \end{array}$$

(12) $$\begin{array}{ccc} & + & \left(N_{0}+t+2\right)\left(H_{N_{0}+t+1}-H_{N_{0}}\right)] \end{array}\;$$

$$\begin{array}{ccc} & = & \frac{\left(N_{0}+t+1\right)}{a_{\mathrm{new}}}[\frac{a_{\mathrm{new}}\frac{Kf_{1}\left(0\right)\left(N_{0}+2\right)}{N_{0}}\left(N_{0}+t+2\right)-2\left(N_{0}+2\right)\left(t+1\right)}{\left(N_{0}+1\right)\left(N_{0}+2\right)} \end{array}$$

(13) $$\begin{array}{ccc} & + & \left(N_{0}+t+2\right)\left\{\psi_{0}\left(N_{0}+t+1\right)-\psi_{0}\left(N_{0}\right)\right\}], \end{array}\;$$

where $N_{0}$ and $Kf_{1}\left(0\right)$ are the initial number of nodes and the initial Kirchhoff index, respectively, and $H_{N}=\sum_{k=1}^{N}k^{-1}$ is the *N*th harmonic number, which can be written as $H_{N}=\gamma +\psi_{0}\left(N+1\right)$, where γ≅0.577 is the Euler–Mascheroni number and $\psi_{0}\left(n\right)=\Gamma^{\prime }\left(n\right)/\Gamma \left(n\right)$ is the digamma function. As its integer argument becomes large, the digamma function satifies $\psi_{0}\left(n\right)\overset{n\to \infty }{\propto }\ln n$. Therefore, when the number of iterations becomes large, the last term in Equation (13) dominates such that

(14) $$\begin{array}{cc} \langle Kf_{1}\left(t\right)\rangle & \overset{t\to \infty }{\propto }N_{t}^{2}\;\log N_{t}. \end{array}$$

**Figure 2:**
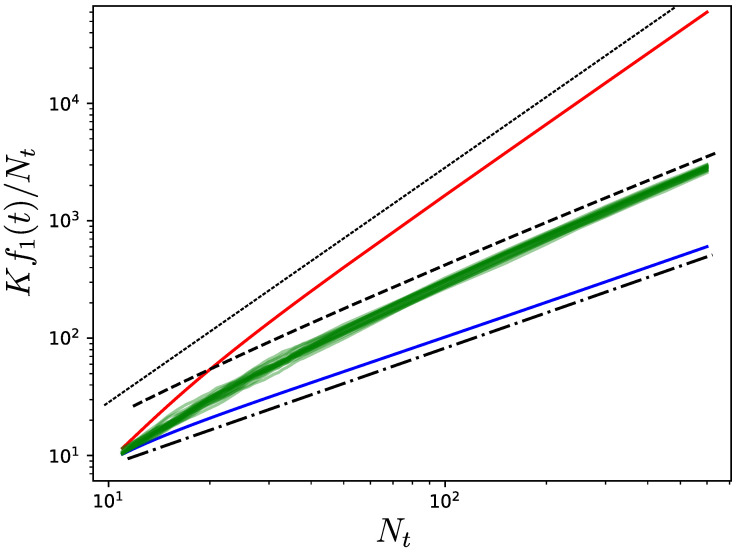
Evolution of the Kirchhoff index divided by the number of nodes $N_{t}$ when a new node is connected to a single existing one at each new iteration. The initial network has ten nodes, and is obtained from a Watts–Strogatz rewiring procedure using nearest neighbors coupling [29]. The green curves correspond to twenty realizations starting from this initial network and recursively adding nodes while selecting the existing nodes to which they connect uniformly and at random. For large $N_{t}$, the green curves follow the scaling of Equation (14). The red and blue curves are obtained by selecting the least and most central existing nodes, respectively, in each iteration. When $N_{t}$ is large, the curves follow the scalings in Equations (16) and (18). The dotted, dashed, and dash-dotted black lines indicate $N_{t}^{2}$, $N_{t}\;\log N_{t}$, and $N_{t}$, respectively.

The scaling is confirmed numerically in Figure 2, where the solid green curves represent twenty realizations of a random growth process starting from ten connected nodes and then adding one new node in each iteration that uniformly connects at random to an existing node. It can be observed that the simulations follow the predicted scaling of Equation (14) provided by the dashed black line. Note that this is the same scaling as in the Wiener index for random recursive trees [13]. This random evolution of the network is bounded by the two limiting cases that we now discuss.

Instead of uniformly picking within the existing nodes, it is possible to instead use a property of the nodes. Here, we discuss what happens when the most or least central node in terms of resistance distance is selected. When the least central node *k* at each iteration *t*, i.e., the one with largest $\sum_{l=1}^{N_{t}}\Omega_{kl}\left(t\right)$, is chosen for connection to the new node, the network tends to form a chain. Therefore, assuming that the weights on the edges are of order 1, when $N_{t}$ becomes large we have

(15) $$\begin{array}{c} \sum_{l=1}^{N_{t}}\Omega_{kl}\left(t\right)≅\frac{N_{t}\left(N_{t}-1\right)}{2}. \end{array}$$

In this case, the Kirchhoff index grows as follows:

(16) $$\begin{array}{ccc} Kf_{1}\left(t+1\right) & ≅ & Kf_{1}\left(t\right)+\frac{N_{t}\left(N_{t}-1\right)}{2}+\frac{N_{t}}{a_{\mathrm{new}}}\overset{t\to \infty }{\propto }N_{t}^{3}, \end{array}$$

which is faster than in the random uniform case in Equation (14). If we instead select the most central node at each time step, then the network becomes star-like. Indeed, by connecting a new node to the most central existing one, its centrality becomes even more important. This means that all of the newly added nodes will connect to the same node. Thus, assuming that the weights of the edges are of order 1, for large $N_{t}$ we have

(17) $$\begin{array}{ccc} \sum_{l=1}^{N_{t}}\Omega_{kl}\left(t\right) & ≅ & \left(N_{t}-1\right), \end{array}$$

(18) $$\begin{array}{ccc} Kf_{1}\left(t+1\right) & ≅ & Kf_{1}\left(t\right)+\left(N_{t}-1\right)+\frac{N_{t}}{a_{\mathrm{new}}}\overset{t\to \infty }{\propto }N_{t}^{2}. \end{array}$$

Interestingly, by selecting the most central node we achieve a scaling for $Kf_{1}$ with $N_{t}$ being only $\log N_{t}$, which is better than in Equation (14), where the node is uniformly chosen from among the existing ones.

#### Discussion

According to Equations (14), (16) and (18), the weakest growth in the Kirchhoff index is obtained when the new nodes simply connect to the most central existing one in terms of resistance distance. Using this mechanism to grow a network leads to a very specific structure in which a single node is connected to almost all the other ones. While such a structure enhances the transient stability of the system by minimizing the growth of the small fluctuations, it makes the system very vulnerable to any failure of this most central node. Indeed, if this node is removed from the system then most of its components become disconnected as well. In light of this structural weakness, selecting nodes at random when adding new nodes seems to be a more robust option; only when the growth of the Kirchhoff index is $\log N_{t}$ is it worth selecting the most central one. Moreover, the connections within the network are more uniformly distributed in the former situation, reducing the number of disconnected components in case of failure. On the other hand side, if it is desirable to increase the fluctuations in the system as much as possible, then new nodes should be connected to the least central node in terms of resistance distance.

### 3.2. One New Node with Two Connections (m=2)

The case in which one node with two edges is added in each new iteration is more complex, as an increasing number of loops is introduced into the network. If the new node is connected to existing nodes *k* and *l*, then the effective resistance along the new path from *k* to *l* is

(19) $$\begin{array}{c} \omega_{kl}=a_{k\;N_{t+1}}^{-1}+a_{l\;N_{t+1}}^{-1}. \end{array}$$

**Figure 3:**
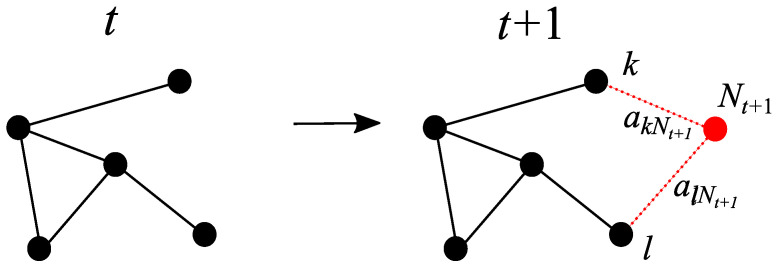
Evolution of the network from iteration *t* to t+1, where a new node (in red) is added that connects to two existing ones *k* and *l* (in black). The label of the new node is $N_{t+1}=N_{t}+1$. In this case, a new path between *k* and *l* is created.

This process is depicted on Figure 3. It is important to remark here that adding node $N_{t+1}$ is not the same as adding an edge between nodes *k* and *l*, which would have a weight of $\omega_{kl}^{-1}$. However, replacing the path on which the new node is located with an equivalent edge provides a lower bound on the new Kirchhoff index. In this way, it is possible to obtain the sum of new resistance distances between the already existing nodes:

(20) $$\begin{array}{ccc} Kf_{k}\left(t+1\right) & = & \frac{1}{2}\sum_{i,j=1}^{N_{t}}\Omega_{ij}\left(t+1\right)+\sum_{i=1}^{N_{t}}\Omega_{iN_{t+1}}\left(t+1\right) \end{array}$$

(21) $$\;\begin{array}{ccc} & = & Kf_{1}\left(t\right)-\frac{\omega_{kl}^{-1}\;\Omega_{kl}^{\left(2\right)}\left(t\right)}{1+\omega_{kl}^{-1}\Omega_{lk}\left(t\right)}+\sum_{i=1}^{N_{t}}\Omega_{iN_{t+1}}\left(t+1\right). \end{array}$$

The variation of the Kirchhoff index is a function of $\Omega_{kl}^{\left(2\right)}\left(t\right)$ and of how central the new node is in terms of resistance distance; see last term in Equation (21). It is challenging to find a closed form expression for the latter term; however, an estimate can be obtained based on the resistance distance in the new network. When the new node is added, the resistance distances between the existing nodes at iteration *t* become

(22) $$\begin{array}{c} \Omega_{ij}\left(t+1\right)=\Omega_{ij}\left(t\right)-\frac{\omega_{kl}^{-1}\;{\left[e_{ij}^{⊤}L^{†}\left(t\right)e_{kl}\right]}^{2}}{1+\omega_{kl}^{-1}\Omega_{kl}\left(t\right)},\;i,j=1,\ldots ,N_{t}, \end{array}$$

where we have replaced the new node with an equivalent edge between *k* and *l* using the Sherman–Morrison–Woodbury formula, as in Equation (9). Using Equation (22), we can approximate the last term in Equation (21) as the weighted average:

(23) $$\begin{array}{ccc} \sum_{i=1}^{N_{t}}\Omega_{iN_{t+1}}\left(t+1\right) & ≅ & \frac{1}{\left(a_{k\;N_{t+1}}+a_{l\;N_{t+1}}\right)}\sum_{j=1}^{N_{t}}\left[a_{kj}\Omega_{kj}\left(t+1\right)+a_{lj}\Omega_{lj}\left(t+1\right)\right]. \end{array}$$

We expect this approximation to be valid when the edge weights surrounding the new node, including $a_{k\;N_{t+1}}$ and $a_{l\;N_{t+1}}$, are homogeneous enough, or when $a_{k\;N_{t+1}}$ and $a_{l\;N_{t+1}}$ are much larger than the surrounding edge weights. When $a_{k\;N_{t+1}}$ and $a_{l\;N_{t+1}}$ are weak, we can expect the centrality of the new node to be lower than that of *k* or *l*. Using this approximation for Equation (21) together with Equation (23) yields the following:

(24) $$\begin{array}{ccc} Kf_{1}\left(t+1\right) & ≅ & Kf_{1}\left(t\right) \end{array}\;$$

$$\begin{array}{ccc} -\frac{\omega_{kl}^{-1}}{1+\omega_{kl}^{-1}\Omega_{kl}\left(t\right)} & \; & \;\left\{\Omega_{kl}^{\left(2\right)}\left(t\right)+\frac{\sum_{j=1}^{N_{t}}\left[a_{k\;N_{t+1}}{\left(e_{kj}^{⊤}L^{†}\left(t\right)e_{kl}\right)}^{2}+a_{l\;N_{t+1}}{\left(e_{lj}^{⊤}L^{†}\left(t\right)e_{kl}\right)}^{2}\right]}{\left(a_{k\;N_{t+1}}+a_{l\;N_{t+1}}\right)}\right\}\\ \; & + & \frac{1}{\left(a_{k\;N_{t+1}}+a_{l\;N_{t+1}}\right)}\sum_{j=1}^{N_{t}}\left[a_{k\;N_{t+1}}\Omega_{kj}\left(t\right)+a_{l\;N_{t+1}}\Omega_{lj}\left(t\right)\right] \end{array}$$

(25) $$\begin{array}{ccc} \; & = & Kf_{1}\left(t\right)-\frac{\omega_{kl}^{-1}}{1+\omega_{kl}^{-1}\Omega_{lk}\left(t\right)}\;\{2\Omega_{kl}^{\left(2\right)}\left(t\right) \end{array}\;$$

$$\;\begin{array}{ccc} \; & + & N_{t}\frac{a_{k\;N_{t+1}}{\left[L_{kk}^{†}\left(t\right)-L_{kl}^{†}\left(t\right)\right]}^{2}+a_{l\;N_{t+1}}{\left[L_{ll}^{†}\left(t\right)-L_{lk}^{†}\left(t\right)\right]}^{2}}{\left(a_{k\;N_{t+1}}+a_{l\;N_{t+1}}\right)}\}\\ \; & + & N_{t}\frac{a_{k\;N_{t+1}}C^{-1}\left(k,t\right)+a_{l\;N_{t+1}}C^{-1}\left(l,t\right)}{\left(a_{k\;N_{t+1}}+a_{l\;N_{t+1}}\right)} \end{array}$$

(26) $$\begin{array}{ccc} \; & = & Kf_{1}\left(t\right)-\frac{\omega_{kl}^{-1}}{1+\omega_{kl}^{-1}\Omega_{lk}\left(t\right)}\;\{2\Omega_{kl}^{\left(2\right)}\left(t\right) \end{array}\;$$

$$\;\begin{array}{ccc} \; & \; & \;+N_{t}\frac{a_{k\;N_{t+1}}{\left[\Omega_{kl}\left(t\right)+C^{-1}\left(k,t\right)-C^{-1}\left(l,t\right)\right]}^{2}+a_{l\;N_{t+1}}{\left[\Omega_{kl}\left(t\right)+C^{-1}\left(l,t\right)-C^{-1}\left(k,t\right)\right]}^{2}}{2(a_{k\;N_{t+1}}+a_{l\;N_{t+1}})}\}\\ \; & + & N_{t}\frac{a_{k\;N_{t+1}}C^{-1}\left(k,t\right)+a_{l\;N_{t+1}}C^{-1}\left(l,t\right)}{\left(a_{k\;N_{t+1}}+a_{l\;N_{t+1}}\right)}\\ \; & = & Kf_{1}\left(t\right)-\frac{\omega_{kl}^{-1}}{1+\omega_{kl}^{-1}\Omega_{lk}\left(t\right)}\;\left\{2\Omega_{kl}^{\left(2\right)}\left(t\right)+N_{t}\frac{\Omega_{kl}^{2}\left(t\right)}{2}\right.\\ \; & + & N_{t}\frac{{\left[C^{-1}\left(k,t\right)-C^{-1}\left(l,t\right)\right]}^{2}}{2}\\ \; & + & \left.N_{t}\frac{\left(a_{k\;N_{t+1}}-a_{l\;N_{t+1}}\right)}{\left(a_{k\;N_{t+1}}+a_{l\;N_{t+1}}\right)}\left[C^{-1}\left(k,t\right)-C^{-1}\left(l,t\right)\right]\Omega_{kl}\left(t\right)\right\} \end{array}$$

(27) $$\begin{array}{ccc} \; & + & N_{t}\frac{a_{k\;N_{t+1}}C^{-1}\left(k,t\right)+a_{l\;N_{t+1}}C^{-1}\left(l,t\right)}{\left(a_{k\;N_{t+1}}+a_{l\;N_{t+1}}\right)} \end{array}\;$$

where we use the relationship between $L_{ii}^{†}$ and the resistance centrality of node *i* (see Equation (7)). This expression provides an approximation of $Kf_{1}\left(t+1\right)$ based only on quantities at iteration *t*. Therefore, in order to reduce the increase of $Kf_{1}$ we should find nodes *k* and *l* such that $\Omega_{kl}^{\left(2\right)}\left(t\right)$ and $\Omega_{kl}\left(t\right)$ are large and have very different resistance centralities, e.g., *k* being part of the most central nodes while *l* belongs to the least central ones. We group together the terms in Equation (27) as follows:

$$\begin{array}{ccc} \mu_{kl}\left(t\right) & = & \frac{\omega_{kl}^{-1}}{1+\omega_{kl}^{-1}\Omega_{lk}\left(t\right)}\left\{2\Omega_{kl}^{\left(2\right)}\left(t\right)+N_{t}\frac{\Omega_{kl}^{2}\left(t\right)}{2}+N_{t}\frac{{\left[C^{-1}\left(k,t\right)-C^{-1}\left(l,t\right)\right]}^{2}}{2}\right. \end{array}$$

(28) $$\begin{array}{ccc} & + & \left.N_{t}\frac{\left(a_{k\;N_{t+1}}-a_{l\;N_{t+1}}\right)}{\left(a_{k\;N_{t+1}}+a_{l\;N_{t+1}}\right)}\left[C^{-1}\left(k,t\right)-C^{-1}\left(l,t\right)\right]\Omega_{kl}\left(t\right)\right\}, \end{array}$$

(29) $$\begin{array}{ccc} \rho_{kl}\left(t\right) & = & N_{t}\frac{a_{k\;N_{t+1}}C^{-1}\left(k,t\right)+a_{l\;N_{t+1}}C^{-1}\left(l,t\right)}{\left(a_{k\;N_{t+1}}+a_{l\;N_{t+1}}\right)}\;. \end{array}$$

**Figure 4:**
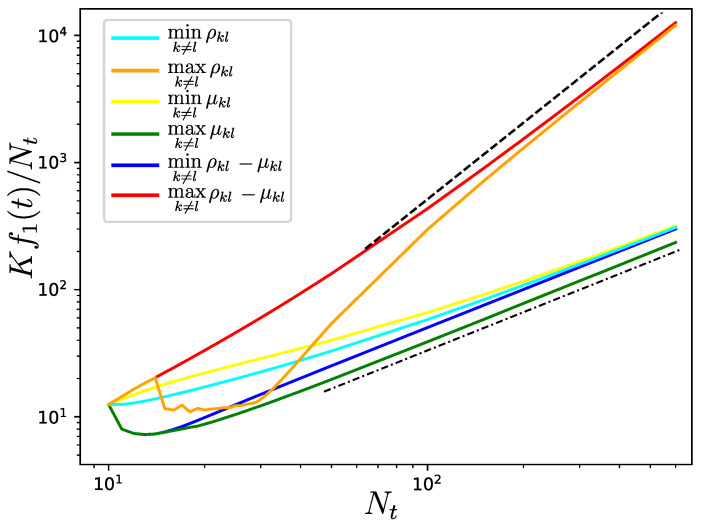
Evolution of the Kirchhoff index divided by the number of nodes $N_{t}$ when a new node is connected to two existing ones (*k* and *l*) in each new iteration. Two nodes are selected by minimizing/maximizing $\mu_{kl}\left(t\right)$, $\rho_{kl}\left(t\right)$, and $\rho_{kl}\left(t\right)-\mu_{kl}\left(t\right)$ for each new iteration. The meaning of each curve is shown in the legend. The initial network has ten nodes, and is obtained from a Watts–Strogatz rewiring procedure with nearest-neighbors coupling [29]. The black dash-dotted and dashed lines show the scalings $N_{t}$ and $N_{t}^{2}$, respectively. Note that in our simulations we ensured that k≠l; however, we found similar scalings when relaxing this condition.

Now, we can choose nodes *k* and *l* that minimize/maximize the latter quantities. More intuitively, we can numerically investigate Equations (28) and (29). In particular, we consider the maximization or minimization at each iteration of $\mu_{kl}\left(t\right)$, $\rho_{kl}\left(t\right)$, and $\rho_{kl}\left(t\right)-\mu_{kl}\left(t\right)$. This is shown in Figure 4. We consider edge weights such that $a_{k\;N_{t+1}}=a_{l\;N_{t+1}}=1$, meaning that the last term in $\mu_{kl}\left(t\right)$ vanishes. As expected, maximizing $\mu_{kl}\left(t\right)+\rho_{kl}\left(t\right)$ (the red curve) in each iteration provides the most important increase in $Kf_{1}\left(t\right)/N_{t}$, which scales as $N_{t}^{2}$. The same scaling is obtained if we maximize only $\rho_{kl}\left(t\right)$ (the orange curve). The minimization of $\mu_{kl}$ (the yellow curve) does not produce a similar increase in the Kirchhoff index, which seems to remain linear, i.e., $Kf_{1}\left(t\right)/N_{t}\propto N_{t}$, as *t* becomes large. Similar linear scaling is observed for the minimization of $\rho_{kl}\left(t\right)-\mu_{kl}\left(t\right)$ (the blue curve) and $\rho_{kl(t)}$ (the cyan curve) as well as for the maximization of $\mu_{kl}\left(t\right)$ (the green curve). Interestingly, it can be observed that the maximization of $\mu_{kl}\left(t\right)$ provides a lower Kirchhoff index than the minimization of $\rho_{kl}\left(t\right)-\mu_{kl}\left(t\right)$; therefore, it is possible to tune the increase of the Kirchhoff index by choosing one or another quantity to optimize at each iteration.

**Figure 5:**
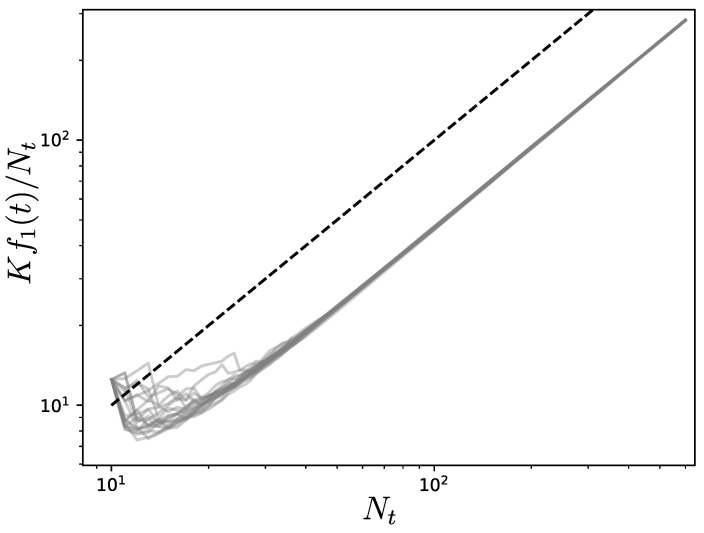
Evolution of the Kirchhoff index divided by the number of nodes $N_{t}$ when a new node is connected to two existing ones at each iteration. The two nodes are selected uniformly at random among the existing ones in each new iteration. Each grey line (twenty in total) is one realization of the process. The initial network has ten nodes and is obtained from a Watts–Strogatz rewiring procedure with nearest-neighbors coupling [29]. The black dashed line shows the linear scaling with $N_{t}$.

Figure 5 shows the simulation results for the case where *k* and *l* are uniformly chosen at random in each new iteration. It can be observed that the twenty realizations of the process yield a linear scaling of $Kf_{1}\left(t\right)/N_{t}$ with $N_{t}$.

#### 3.2.1. Discussion

In Section 3.1, we have seen that selecting the existing node to which the new one connects uniformly and at random is more worthwhile compared to selecting the most central one when the growth of the Kirchhoff index is $\log N_{t}$. Interestingly, when the new nodes connect to two existing ones, selecting them uniformly at random produces the same scaling as when minimizing the relevant quantity $\rho_{kl}\left(t\right)-\mu_{kl}\left(t\right)$. Therefore, when growing a network by connecting the new node to two existing ones, to achieve the best scaling it is only necessary to ensure that the nodes are selected uniformly and at random. Of course, the latter is only true as long as the approximation in Equation (23) holds. If the goal is to disrupt the system, a scaling of the Kirchhoff index $N_{t}$ is worthwhile compared to the previous situation, and is obtained by maximizing either $\rho_{kl}\left(t\right)-\mu_{kl}\left(t\right)$ or $\rho_{kl}\left(t\right)$. The latter can be achieved by choosing nodes that are close in terms of $\Omega_{kl}\left(t\right)$ and $\Omega_{kl}^{\left(2\right)}\left(t\right)$ while being rather peripheral in the network, i.e., small C(k,t) and C(l,t).

#### 3.2.2. Remark

It is important to be careful when interpreting Equations (21) and (24), as well as to note that on average the Kirchhoff index increases at least linearly with $N_{t}$, as can be seen from Equation (8). More intuitively, in the case with $m=N_{t}$ (meaning that the number of edges added in each iteration grows with the system size), when assuming an initial all-to-all network we have

(30) $$\begin{array}{c} Kf_{1}\left(t+1\right)=N_{t}, \end{array}$$

which increases monotonically. In this situation, as many new paths as possible should be added between the existing nodes when introducing a single new node. Therefore, the Kirchhoff index must increase for any $m<N_{t}$. In Equation (21), we might instead reduce the amplitude of the increase, or sometimes even decrease $Kf_{1}$ by carefully selecting *k* and *l*; however, this can only remain true for a few iterations.

## 4. Conclusions

In this paper, we have considered the evolution of random networks in which a new node is added in each new iteration and connected to one or two existing nodes. When the new nodes are only connected to a single existing node, the scaling of $Kf_{1}\left(t\right)/N_{t}$ with the number of nodes is between $N_{t}$ and $N_{t}^{2}$ as the number of iterations becomes large. When the existing node is randomly and uniformly chosen, the scaling is only logarithmically worse than the lower bound, i.e., $Kf_{1}\left(t\right)/N_{t}\overset{t\to \infty }{\propto }N_{t}\;\log N_{t}$. In the more complex situation in which the new nodes are connected to two existing nodes, a recursive expression is derived for the evolution of $Kf_{1}\left(t\right)$. The latter is essentially provided by $\rho_{kl}\left(t\right)-\mu_{kl}\left(t\right)$, which can be expressed in terms of the resistance distances and centralities, i.e., $\Omega_{kl}$, $\Omega_{kl}^{\left(2\right)}$, $C^{-1}\left(k,t\right)$, and $C^{-1}\left(l,t\right)$; see Equations (28) and (29). We show that by introducing a bias in the selection of *k* and *l* towards the minimum/maximum of these quantities, it is possible to tune the increase of $Kf_{1}\left(t\right)/N_{t}$ from linear to quadratic in $N_{t}$. For m>2, it is much more challenging to obtain analytical expression for the evolution of $Kf_{1}$. The same applies to the case in which *m* is a function of the number of nodes. Nonetheless, using the lower bound in Equation (8) allows the minimal scaling of the Kirchhoff index to be obtained by correctly choosing $n_{e}\left(N\right)$.

The scenario we have considered here applies to evolving systems in which a single new node is added at each iteration and connects to existing nodes. This can represent situation such as a new molecule forming bonds with another group of molecules, or an autonomous vehicle joining a platoon by interacting with one or many of its members. Using the results presented here, it is possible to anticipate the scaling of the Kirchhoff index based on how new units connect to the existing ones. Thus, our results provide insights into the evolution of fluctuations in networked systems such as consensus dynamics and synchronized systems that are diffusively coupled.

### Outlook

In this manuscript, we have considered two fundamental mechanisms for growing a networked systems: (i) adding edges to an existing system, and (ii) adding nodes that connect to one or two existing units in the system. We investigated these two scenarios independently, finding that different scalings for the Kirchhoff index are achievable. In order to describe realistic systems such as swarm formation in groups of animals or autonomous robots, it is necessary to consider both of these scenarios, with one potentially occurring immediately after or even simultaneously with the other. Future research should consider the extension of our results to cases in which multiple connected nodes are added at the same time. Additionally, future research could investigate how other properties are modified by the growth of the network. Notably, the Kirchhoff index is directly related to the small fluctuations of networked oscillators; however, other system characteristics, such as the ability of a network to synchronize, typically depend on the maximum and minimum eigenvalues of the Laplacian matrix.

## Data Availability

The data presented in this study are available upon requeset from the corresponding author.
